# Is Re-introducing Major Open and Minimally Invasive Surgery during COVID-19 Safe for Patients and Healthcare Workers? An International, Multi-centre Cohort Study in the Field of Oesophago-gastric Surgery

**DOI:** 10.1245/s10434-021-09885-0

**Published:** 2021-04-17

**Authors:** Mohamed Alasmar, Afsana Kausar, Alexander Berend-Jan Borgstein, Johnny Moons, Sophie Doran, Stefano de Pascale, Rafael Restrepo, Apollonia Verrengia, Mariella Alloggio, Ana Moro Delgado, Sacheen Kumar, Ismael Díez del Val, Simone Giocapuzzi, Gian Luca Baiocchi, Marta de Vega Irañeta, Gabriel Salcedo, Peter Vorwald, Uberto Fumagalli Romario, Philippe Nafteux, Suzanne Gisbertz, Mohammed Asif Chaudry, Bilal Alkhaffaf

**Affiliations:** 1 Department of Oesophago-Gastric Surgery, Salford Royal NHS Foundation Trust, Salford Royal Hospital, Manchester, UK; 2 Division of Cancer Sciences, School of Medical Sciences, Faculty of Biology, Medicine and Health, University of Manchester, Manchester, UK; 3 Department of Surgery, Cancer Center Amsterdam, Amsterdam UMC, University of Amsterdam, Amsterdam, The Netherlands; 4 Department of Thoracic Surgery, University Hospitals Leuven, Leuven, Belgium; 5 Department of Academic Surgery, Royal Marsden NHS Foundation Trust, London, UK; 6 Istituto Europeo di Oncologia – IRCCS, Milan, Italy; 7 Hospital Universitario Fundación Jiménez Diaz, Madrid, Spain; 8 Department of Clinical and Experimental Sciences, University of Brescia, Brescia, Italy; 9 University of Verona, Verona, Italy; 10 Basurto University Hospital, Bilbao, Spain; 11 Hospital Universitario Fuenlabrada, Madrid, Spain

## Abstract

**Introduction:**

The COVID-19 pandemic has resulted in unparalleled changes to patient care, including the suspension of cancer surgery. Concerns regarding COVID-19-related risks to patients and healthcare workers with the re-introduction of major complex minimally invasive and open surgery have been raised. This study examines the COVID-19 related risks to patients and healthcare workers following the re-introduction of major oesophago-gastric (EG) surgery.

**Patients and Methods:**

This was an international, multi-centre, observational study of consecutive patients treated by open and minimally invasive oesophagectomy and gastrectomy for malignant or benign disease. Patients were recruited from nine European centres serving regions with a high population incidence of COVID-19 between 1 May and 1 July 2020. The primary endpoint was 30-day COVID-19-related mortality. All staff involved in the operative care of patients were invited to complete a health-related survey to assess the incidence of COVID-19 in this group.

**Results:**

In total, 158 patients were included in the study (71 oesophagectomy, 82 gastrectomy). Overall, 87 patients (57%) underwent MIS (59 oesophagectomy, 28 gastrectomy). A total of 403 staff were eligible for inclusion, of whom 313 (78%) completed the health survey. Approaches to mitigate against the risks of COVID-19 for patients and staff varied amongst centres. No patients developed COVID-19 in the post-operative period. Two healthcare workers developed self-limiting COVID-19.

**Conclusions:**

Precautions to minimise the risk of COVID-19 infection have enabled the safe re-introduction of minimally invasive and open EG surgery for both patients and staff. Further studies are necessary to determine the minimum requirements for mitigations against COVID-19.

**Electronic supplementary material:**

The online version contains supplementary material available at (10.1245/s10434-021-09885-0) contains supplementary material, which is available to authorized users.

The COVID-19 pandemic has seen unprecedented changes to the provision of healthcare so that services can focus their efforts on managing the crisis. Due to significant concerns pertaining to the safety of surgery and the associated increased morbidity and mortality, many elective operative programmes were suspended.^1^ Following the first ‘peak’ of the pandemic, many regions began the re-introduction of elective surgery on a priority basis. Cancer surgery was high on this priority list.

Despite significant improvements in approaches to peri-operative care over the last decade, surgery for oesophago-gastric (EG) cancer is still associated with significant morbidity.^2^–^4^ Whilst wide-ranging mitigations for COVID-19 have become commonplace, the re-introduction of EG surgery has rightly highlighted concerns. Despite their need for life-saving treatments, patients remain fearful about their risk of contracting COVID-19 in hospital.^5^,^6^ In addition to the increased risk posed to patients, there may also be an unquantified risk to medical staff involved in operative cases where the abdominal and thoracic cavities are exposed for long periods.^7^,^8^ As a result, some centres have been reluctant to re-start minimally invasive surgery (MIS) programmes because of the perceived risks of the escape of aerosolised COVID-19 viral particles from the abdominal and thoracic cavities under high pressure.

Proponents of MIS argue that these risks are not evidence based and can be easily mitigated with the use of adequate personal protective equipment (PPE). Whilst healthcare services have been keen to ensure that adequate PPE is available for staff, it is not known what the minimum necessary requirements for PPE are in the context of COVID-19. Furthermore, in centres where the provision of MIS was commonplace, suspending these approaches may be exposing patients to wound and respiratory complications, which would result in longer lengths of stay in hospital. This would be particularly disadvantageous at a time where the risk of contracting COVID-19 in hospital may be significant.^9^

This study aims to assess, in the context of significant regional levels of COVID-19, the safety of re-introducing MIS and open surgery for EG disease, both from the perspective of the patient and healthcare workers. The objectives are as follows:

- To determine current practice with respect to mitigations aimed at reducing the risks of COVID-19 amongst patients undergoing EG surgery and healthcare workers involved in their care.
- In the context of these mitigations, to determine the incidence of COVID-19 and non-COVID-19 morbidity and mortality in both MIS and open surgery for EG cancer surgery.
- In the context of these mitigations, to determine the risk of ‘patient-to-staff’ transmission of COVID-19 amongst healthcare workers involved in the operative care of EG surgical patients.

## Patients and Methods

### Study Design

This was an international, multi-centre, observational study of patients who were scheduled for elective minimally invasive or open oesophagectomy or gastrectomy. To assess the potential risk to healthcare workers of undertaking these procedures, all staff members who were present in theatre at the time of surgery were asked to complete an anonymous COVID-related health questionnaire.

### Setting

Participant data were collected from nine specialist European centres for EG surgery. Each centre served patients from populations that had been particularly affected by the COVID-19 pandemic from the perspective of infections and deaths (UK, Italy, Spain, Belgium and the Netherlands).

### Participants

Consecutive patients who had undergone EG surgery between the 1 May 2020 and the 1 July 2020 at each centre were included. Patients were followed up for a minimum of 30 days. The following eligibility criteria for patients were applied:

- Aged 18 years and over
- Procedure: oesophagectomy or gastrectomy (partial or total)
- Pathology: malignant and benign disease
- Operative approach: totally minimally invasive, hybrid minimally invasive or totally open surgery

All healthcare workers involved in the care of the patient within the operating theatre were invited to complete an anonymous health survey (Supplementary Appendix 1). This group was the focus of our survey as they were deemed at particular risk from potential ‘patient-to-staff transmission’ due to their involvement in aerosol generating procedures (intubation, extubation, minimally invasive surgery and thoracic surgery). Local collaborators completed a register of eligible staff members during each case to ensure all eligible healthcare workers could be contacted to complete the survey. This non-validated questionnaire was developed by the study team with the objective of identifying the incidence of COVID-19 in medical staff involved in the care of included patients. Surveys were sent out after 15 July 2020 (14 days after the final included patient underwent surgery) to accommodate for a COVID-19 incubation period of up to 2 weeks.^10^ Regular weekly reminders were sent out for a period of 4 weeks to ensure a survey response rate of at least 70% was achieved.

### Procedures

Laboratory testing for COVID-19 was based on viral RNA detection by quantitative reverse transcription polymerase chain reaction (RT-PCR). Sampling, including nasopharyngeal swabs and bronchoalveolar lavage, and analyses were undertaken according to local hospital protocols. RT-PCR testing was available in all participating centres. Oesophagectomy included both two- (intra-thoracic anastomosis) and three-stage (cervical anastomosis) approaches. Totally minimally invasive oesophagectomy (tMIE) was defined as surgery using laparoscopic and thoracoscopic techniques, with hybrid minimally invasive oesophagectomy (hMIE) defined as surgery using a laparoscopic approach with open thoracotomy. Both total and partial gastrectomy requiring alimentary reconstruction were included, however, wedge excision of gastric lesions was excluded from analysis.

### Variables

Data were collected prospectively by each local collaborating team using a standardised Microsoft Excel spreadsheet. Patient demographics (age, sex, performance status, ASA grade and Charlson co-morbidity index), disease data (histology, disease stage and neo-adjuvant therapy), COVID-19-related variables (previous RT-PCR testing and results) and operative approach were collected for each case.

Participating centres were also asked to describe local precautions employed to reduce the risk of COVID-19 to both patients and staff (e.g. patient and staff screening or testing, patient flow in hospital and intra-operative mitigations). In addition, data from the European Centre for Disease Prevention and Control (https://www.ecdc.europa.eu/) were collected to describe COVID-19-related hospital and intensive care unit occupancy and death before and during the study period.^11^ This was the preferred method of contextualising our findings due to the significant limitations associated with testing in the first wave of the pandemic.

### Outcomes

The primary patient outcome was 30-day COVID-19-related mortality (confirmed by RT-PCR test) with the day of surgery defined as day 0. Secondary outcomes were COVID-19 infection (confirmed by RT-PCR test), non-COVID-19-related respiratory complications and other complications as defined by established international guidelines (www.esodata.org and www.gastrodata.org) in the field of EG surgery.^12^,^13^ Severity of outcomes was graded according to the Clavien–Dindo (CD) scale and Comprehensive Complications Index (CCI).^14^,^15^ The primary outcome from the healthcare worker survey was the incidence of COVID-19 infection.

### Study Size

A sample size was not applicable to this study. Whilst recruiting centres were defined as ‘high volume’ in comparison with others across Europe (at least 50–100 major EG cases per annum), it was necessary to balance this against the likely lower operative volumes as a result of the pandemic. Our aim was to produce relevant and externally valid evidence within a relatively short period of time, and so a pragmatic target of at least 100 eligible cases was set by the study management team.

### Data Sources

Only routine, anonymised patient data were collected with no change to clinical pathways.

### Data Analysis

Descriptive statistics were used to report participant characteristics and outcomes in this study including mean, standard deviation and 95% confidence intervals where appropriate. Patients outcomes were grouped according to surgery type (oesophagectomy or gastrectomy) and operative approach (open or minimally invasive surgery). Hybrid minimally invasive oesophagectomy (e.g. laparoscopic abdomen and open thoracotomy) was included in the minimally invasive group. Statistical analyses of the present study were performed using the R statistical package (R Foundation for Statistical Computing, Vienna, Austria. https://www.R-project.org/).

## Results

### Overview

**Fig. 1:**
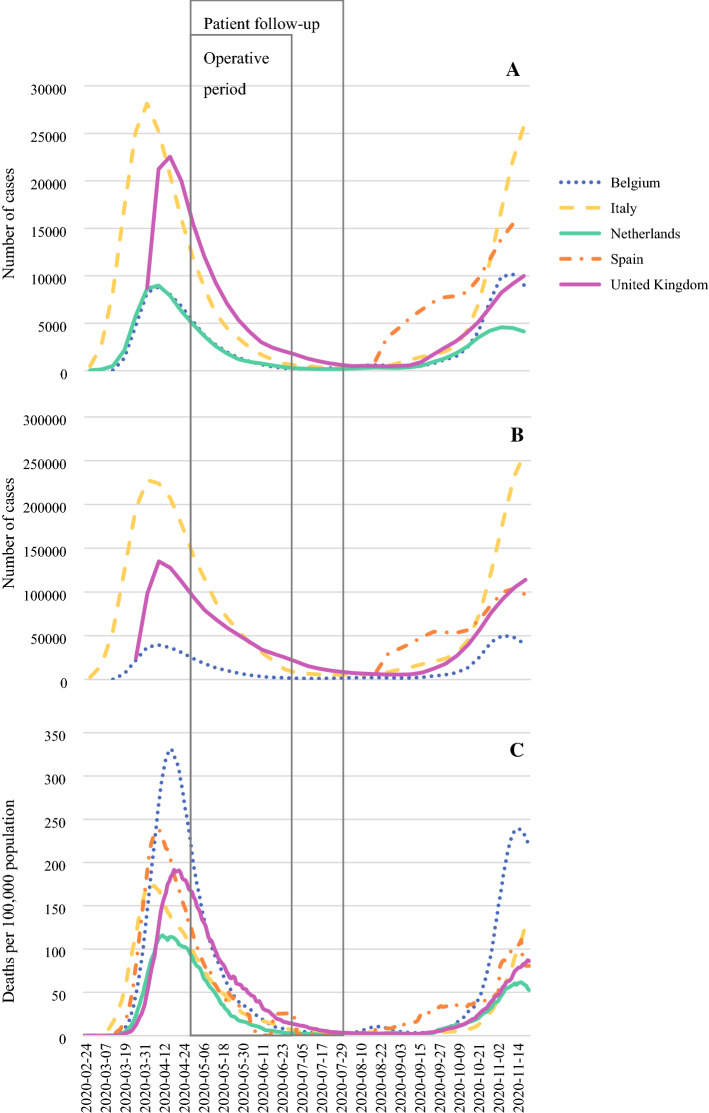
Population impacts of COVID-19 during our study period on **A** intensive care occupancy, **B** hospital occupancy and **C** deaths

A total of 158 patients and 403 healthcare workers were eligible for inclusion into the study. Figure 1 shows the weekly incidence of COVID-19-related impacts in the regional populations served by participating centres since the start of the pandemic. Table 1 presents the precautions taken by centres to reduce the COVID-19-related health risk amongst patients and healthcare workers. No centre mirrored another with respect to mitigations across all domains (hospital precautions, patient screening, staff screening and protection, and intra-operative precautions).

**Table 1:**
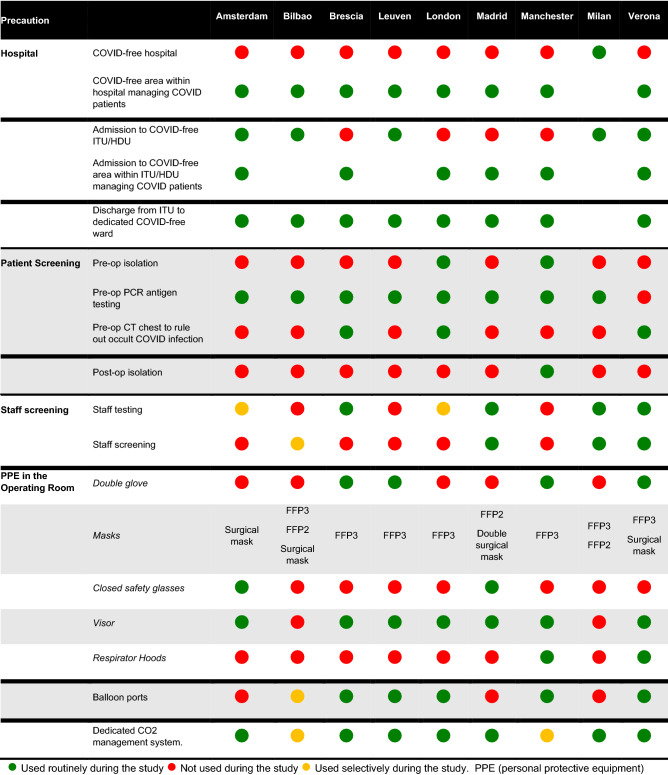
Precautions taken to minimise the risk of COVID-19 infections amongst patients and medical staff

**Table 2:** Characteristics of patient participants

	*n* = 158 (%)
*Demographics*	
Age (years)	64.5 (mean) (SD 10.80)
Sex	
Male	108 (68.35%)
Female	50 (31.65%)
Charlson co-morbidity index	4 (mean) (SD 2.12)
WHO performance status	
0	84 (53.16%)
1	57 (36.08%)
2	12 (7.59%)
3	2 (1.27%)
Unknown	3 (1.27%)
ASA	
1	10 (6.33%)
2	92 (58.23%)
3	52 (32.91%)
4	4 (2.53%)
*Disease*	
Benign	13 (8.23%)
Malignant	145 (91.77%)
Malignant subtype	145
Adenocarcinoma	112 (77.2%)
Squamous cell carcinoma	16 (11%)
GIST	9 (6.2%)
Others	7 (4.8%)
Unknown	1 (0.7%)
Cancer stage	128
1	15 (11.72%)
2	45 (38.28%)
3	51 (39.84%)
4	9 (7.03%)
Unknown	8 (6.25%)
Neoadjuvant therapy	
Chemotherapy	64 (40.51%)
Chemoradiotherapy	39 (24.68%)
Surgery alone	55 (34.81%)

*SD* standard deviation

### Patient Outcomes

A summary of the 158 eligible patient characteristics is presented in Table 2. A total of 71 oesophagectomies and 82 gastrectomies were completed. Three cases (1.9%) were abandoned, and two (1.3%) gastrectomy were converted to palliative bypass due to metastatic disease. A total of 67 (42.4%) open procedures, 88 (55.7%) minimally invasive (71 totally minimally invasive, 16 laparoscopy and open thoracotomy and 1 laparotomy with thoracoscopy) and 3 (1.9%) minimally invasive converted to open procedures were undertaken. Data completeness with at least 30-day follow-up was achieved in all cases. Primary and secondary outcomes for each operative approach of the 153 completed cases are summarised in Table 3. Supplementary Appendix 1 provides a comprehensive anonymised report of all patient outcomes by centre as defined by established international guidelines in the field of EG surgery.^12^,^13^

**Table 3:** Outcomes of patients undergoing oesophago-gastric resectional surgery between 1 May and 31 June 2020

	All cases	Oesophagectomy			Gastrectomy		
	*n* = 158	All *n* = 71	Open *n* = 12	Minimally invasive *n* = 59	All *n* = 82	Open *n* = 54	Minimally invasive *n* = 28
Complications (any grade) (%)	94 (59.49%)	49 (69.01%)	9 (75.00%)	40 (67.80%)	45 (54.88%)	37 (68.52%)	8 (28.57%)
Worst Clavien–Dindo grade							
1 (%)	21 (13.29%)	4 (5.63%)	2 (16.67%)	2 (3.39%)	17 (20.73%)	14 (25.93%)	3 (10.71%)
2 (%)	44 (27.85%)	24 (33.80%)	5 (41.67%)	19 (32.20%)	20 (24.39%)	17 (31.48%)	3 (10.71%)
3 (%)	16 (10.13%)	10 (14.08%)	1 (8.33%)	9 (15.25%)	6 (7.32%)	4 (7.41%)	2 (7.14%)
4 (%)	12 (7.59%)	11 (15.49%)	1 (8.33%)	10 (16.95%)	1 (1.22%)	1 (1.85%)	0
5 (%)	1 (0.63%)	0	0	0	1 (1.22%)	1 (1.85%)	0
Mean CCI	15.6	23.0	23.8	22.9	9.3	11.5	5.0
Standard deviation	*19.3*	*20.5*	*20.8*	*20.4*	*16.4*	*17.9*	*12.2*
95% confidence interval	*12.52*–*18.59*	*13.14*–*32.89*	*6.24*–*43.10*	*7.71*–*38.01*	*5.30*–*13.30*	−*3.76*–*26.79*	−*1.81*–*11.88*
COVID-19 infection							
Not tested (%)	116 (73.42%)	56 (78.87%)	11 (91.67%)	45 (76.27%)	60(73.17%)	38 (70.37%)	22 (78.57%)
Tested negative (%)	37 (23.41%)	13 (18.31%)	1 (8.33%)	14 (23.73%)	22 (26.83%)	16 (29.63%)	6 (21.43%)
Tested positive (%)	0	0	0	0	0	0	0
Respiratory complications	55 (34.81%)	37 (52.11%)	5 (41.67%)	32 (54.24%)	18 (21.95%)	13 (24.07%)	5 (17.86%)
Pneumonia (%)	24 (15.19%)	12 (16.90%)	2 (16.67%)	10 (16.95%)	12 (14.63%)	7 (12.96%)	5(17.86%)
Pneumothorax (%)	5 (3.16%)	4(5.63%)	0	4(6.78%)	1 (1.22%)	1 (1.85%)	0
Pleural effusion requiring additional drainage procedure (%)	15 (9.49%)	13 (18.31%)	3 (25.00%)	10 (16.95%)	2 (2.44%)	2 (3.70%)	0
Acute aspiration (%)	2 (1.27%)^a^	2 (2.82%)	0	2 (3.39%)	0	0	0
Chest tube drainage for > 10 days post-op (%)	1 (0.63%)	1 (1.41%)	0	1 (1.69%)	0	0	0
Respiratory failure requiring re-intubation (%)	8 (5.06%)	5 (7.04%)	0	5 (8.47%)	3 (3.66%)	3 (5.56%)	0

^a^One patient who had their procedure abandoned suffered this complication

*CCI* comprehensive complication index

Pre-operative COVID-19 testing was undertaken in 149 patients (94%), with one centre not adopting a routine pre-operative testing policy at the time of the study. A total of 2 of the 149 tested (1.3%) were found to be positive for COVID-19, resulting in the postponement of their surgery. One patient tested negative 2 weeks later, whilst the other required multiple tests over the course of 6 weeks before a negative COVID-19 test was achieved. Both proceeded with surgery with curative intent as planned.

With respect to the primary outcome, 39 (24.7%) patients underwent post-operative RT-PCR testing for suspected COVID-19 infections, of which none were positive. One death was reported (0.6%), which was ascribed to respiratory failure following open total gastrectomy in a patient whose post-operative RT-PCR test for COVID-19 was negative. Median length of stay in hospital was 10 days (oesophagectomy 12 days, gastrectomy 8 days), with 153 (97.5%) being discharged home, and the remaining 4 (2.5%) to an intermediate care facility.

### Healthcare Survey Outcomes

Of the 403 healthcare workers eligible for inclusion into this study, 313 (77.7%) completed the COVID-19-related health survey (characteristics and outcomes summarised in Table 4). All centres had access to RT-PCR testing for staff members suspected of COVID-19. With respect to the primary outcome, two (0.6% of total responses) healthcare workers (one surgeon, one scrub nurse) from the same hospital tested positive for COVID-19 during the study period. Both had participated in fewer than five gastrectomies, no oesophagectomies and no minimally invasive surgery. Both participants reported that household members had shown symptoms of COVID-19 and/or tested positive.

**Table 4:** Characteristics of healthcare workers who completed COVID-19-related health survey

	*n *= 313
*Job title*	
Anaesthetic support staff	23 (7.4%)
Anaesthetists	68 (21.7%)
Surgeon	96 (30.7%)
Scrub nurse	97 (31.0%)
Other theatre team	17 (5.4%)
Other	12 (3.3%)
*Days worked in theatre*	
< 10	26 (8.3%)
10–20	54 (17.3%)
21–30	55 (17.6%)
31–40	53 (16.9%)
> 40	125 (39.9%)
*Number of EG surgeries participated in*	
< 5	181 (57.8%)
5–10	76 (24.3%)
11–15	25 (8.0%)
16–20	15 (4.8%)
> 20	16 (5.1%)
*Number of non*-*EG surgeries participated in*	
0	9 (2.9%)
< 5	42 (13.4%)
5–10	37 (11.8%)
11–15	33 (10.5%)
16–20	42 (13.4%)
> 20	150 (47.9%)
*COVID*-*19*-*related information*	
Required to isolate prior to study	50 (16.0%)
Tested for COVID-19 prior to study	124 (39.6%)
Negative	106 (33.9%)
Positive	18 (5.75%)
Required to isolate or be tested during study	40 (12.78%)
Negative	38 (12.1%)
Positive	2 (0.6%)
Suspected or confirmed positive members of household	19 (6.0%)

## Discussion

This study investigated the re-introduction of open and minimally invasive gastrectomy and oesophagectomy during the first wave of the COVID-19 pandemic. Our results suggest that, despite significant levels of COVID-19 in local populations, mitigations against the risk of COVID-19 infection were sufficient to safely re-introduce major EG surgery across Europe. Furthermore, concerns surrounding the use of MIS have not been substantiated in our multi-centre cohort, suggesting that laparoscopic and thoracoscopic surgery can continue without risk to patients or healthcare workers in the operating theatre. Patients were generally older, suffered from co-morbidities and would potentially be exposed to devastating consequences had they contracted COVID-19.^1^ Furthermore, the centres included in this study served populations particularly affected by COVID-19. Hence, whilst our case study focused on patients undergoing major EG surgery, the results are likely applicable to many patient groups and provide data that can be used to reassure both patients and healthcare workers.

Approaches to minimise the risk of developing COVID-19 in both patients and staff undoubtedly contributed to our findings. However, these approaches were not uniform or standardised and reflect a lack of evidence base and differing local, national and international responses from governments and professional societies. Understanding what constitutes ‘minimum required precautions’ is a topic which requires further exploration. As a minimum, all patients in our study were managed in ‘COVID-19-free’ areas within hospitals and most were tested pre-operatively without needing pre- or post-operative isolation. The greatest levels of variation seemed to relate to the level of PPE worn by staff in theatre. Whilst necessary, precautions must be carefully balanced against unintended consequences such as the devastating impact on surgical waiting lists which may now take years to rectify.^16^,^17^ For example, policies that require both patients and their households to self-isolate before major elective surgery are simply impractical for most, potentially psychologically harmful and may in fact hinder the efficient utilisation of scarce operating theatre capacity. In our study, most centres did not require patients to isolate pre-operatively. Whilst it is possible that a proportion of patients may have initiated a form of isolation or ‘social distancing’, our data suggest that this does not need to be prescriptive. Furthermore, others have suggested that the use of some PPE may be associated with significant challenges during intra-operative communication between staff members.^18^ As local, regional and national PPE recommendations evolve, careful evaluation will be required to ensure that patients and staff remain safe. Whilst it should be recognised that the local population incidence of COVID-19 will play a factor, we recommend that further evidence-based guidance from national and international professional societies be developed so that guidance can be updated.

In the current climate, minimising the direct risks of COVID-19 to patients undergoing major complex surgery is paramount. However, as EG surgery is associated with significant risk of complications, a ‘safe’ surgical pathway for this patient group must also ensure that appropriate resources are available to manage morbidity in the post-operative period. Our study suggests that collaborators were able to deliver safe care to patients and achieve low levels of morbidity and mortality. However, many healthcare services have ‘re-deployed’ vitally important members of surgical, anaesthetic and nursing teams and diverted intensive care resources to help manage the pandemic. This leaves elective surgical services vulnerable and has led to large numbers of elective cancellations.^17^ Many centres included in our study had suspended their surgical programmes during the initial peak incidence of the pandemic for this reason. Some collaborators adopted different approaches and rationalised regional services so that high-risk surgery was undertaken on ‘cold operative sites’ where patients with COVID-19 were not admitted. The lead collaborating centre in our study aimed to limit hospital occupancy to around 60% during the first wave so that resources could be appropriately shared between COVID-19, emergency and cancer surgery patients. Such figures are not necessarily applicable to other centres, which must take into account the number of complex/major surgical services within the hospital, the local population levels of COVID-19 and available resources.

One of the unintended consequences of the pandemic has been the devastating impact on patients with disease unrelated to COVID-19. Several guidelines that detail how surgical care should be prioritised during this time have been developed for clinical practice.^19^,^20^ Whilst delaying cancer surgery would understandably risk the repercussions of disease progression, many other patients with conditions that impact severely on quality of life have also been affected. Most collaborating centres only undertook cancer surgery when elective programmes first recommenced. However, as confidence grew that patients could be treated safely, a small number of surgical cases for benign disease were successfully undertaken. The argument for undertaking (complex) benign elective cases during the pandemic is one which should be considered alongside local resource availability and therefore broad recommendations cannot be made. Whilst it is understandable that this subset of patients will be prioritised differently from cancer cases, the results of delays in this cohort should not be ignored.

The background incidence of COVID-19 in the local population is a key consideration when reflecting on this study’s findings. We opted to describe the impact of COVID-19 in terms of hospitalisations, intensive care unit bed occupancy and deaths as testing capabilities were significantly limited during the first wave of the pandemic. This enables more reliable comparisons between the first pandemic wave to be made with subsequent waves, allowing healthcare professionals and managers to use previous experience, aid decision-making and service organisation. Collaboration was purposefully sought from centres serving populations significantly impacted by the pandemic. Healthcare services, medical staff and patients can therefore be reassured that our findings are likely to be widely applicable. Furthermore, whilst we included patients from regions which had seemingly past their ‘peak’ incidence, the COVID-19 prevalence remained significant during our operative period. Nonetheless, all the regions included in this study have since been through additional surges of COVID-19 cases, and it is unquestionable that, at the time of writing, we are in a second, and in some cases third, wave of the pandemic. It is possible that, as some regions surpass the regional COVID-19 prevalence which occurred during our study, and despite robust mitigations, COVID-19 infections may begin to appear in patients undergoing major complex surgery with devastating impacts. Recent estimates from the World Health Organization suggest that populations will remain at risk for at least the next 2 years.^21^ It is therefore essential to establish robust systems which will provide the safe treatment of complex life-shortening or life-changing disease for the foreseeable future and ensure that outcomes continue to be monitored and transparently reported.

It has been suggested that MIS may expose medical staff to increased risk of contracting COVID-19 infection due to the possibility of virus aerosolisation in surgical smoke.^7^,^8^ In addition, it is generally accepted that operative times are longer for MIS compared with open surgery, particularly in the field of EG disease, potentially extending the viral exposure. Initially, these concerns resulted in guidance based on low-level evidence advising healthcare services to avoid MIS where possible.^22^ Many groups have now updated their recommendations with less cautionary language. Nonetheless, the initial guidance meant that some patients could not be offered surgical approaches, which, particularly in the case of EG surgery, can lead to fewer complications and a shorter length in hospital stay.^23^–^25^ Two healthcare workers developed self-limiting COVID-19 infections during the study period. Both had participated in fewer than five open gastrectomy surgeries with no involvement in either MIS or oesophagectomy. Both reported that household members had exhibited symptoms and/or tested positive for COVID-19, suggesting that their cases were not nosocomial. Our findings therefore support the continued use of MIS on the proviso that risk-reducing precautions are maintained. Again, what these precautions should entail is a matter which requires further study. For example, whilst recruiting centres did not uniformly use balloon ports to reduce the risk of smoke escape, all used some form of dedicated smoke evacuation filter.

The strengths of this study include its prospective, multi-centre design. We considered the safety of not only patients, but also medical staff, of whom 77% completed their health survey. To the best of the authors’ knowledge, this is the first study in this field examining the re-introduction of major complex EG surgery across several centres during the COVID-19 pandemic. Furthermore, our study considered the background incidence of COVID-19 in the local populations of each participating centre and its relation to the first pandemic ‘peak’.

There are some limitations which require further discussion. We adopted a pragmatic approach to measuring the incidence of COVID-19 amongst healthcare workers. We opted to invite those who were involved in the operative care of patients to complete the survey. This was because the study aimed partly to provide data about the safety of minimally invasive surgery to staff. It could be argued that other healthcare workers, such as nurses, may similarly be at increased risk from patient-to-staff transmission. The anonymous nature of our health survey aimed to encourage all types of medical staff, some of whom had never previously participated in research, to engage in an open and transparent manner. However, such an approach relies purely on self-reporting, which is associated with inherent limitations. For example, the survey would not establish a reliable understanding of how many healthcare workers had contracted COVID-19 and remained asymptomatic, as not all the recruiting centres adopted regular testing for their employees. The rate of asymptomatic COVID-19-positive healthcare workers will vary from location to location and is also likely to change as the pandemic progresses.^26^ And, whilst the risk of nosocomial infection between healthcare workers is likely to be low,^27^ there remains a possibility of in-hospital transmission which mandates the continued need for PPE. Furthermore, medical staff involved in the peri-operative care of patients often worked across numerous specialities. Had the COVID-19 infection rates amongst staff been significant, it would have been difficult to discern whether this was due to a particular type of surgery or indeed whether the infection was acquired outside of the hospital environment. However, given the extremely low incidence of COVID-19 amongst staff, we do not believe that this influenced the findings of our study. Finally, it is accepted that establishing accurate population incidence of COVID-19 is difficult.^25^ Moreover, the accuracy of laboratory testing used to ascertain whether patients contracted COVID-19 is associated with its own challenges.^28^ As such, we acknowledge that these factors may have impacted on the findings presented in this study.

## Conclusions

Major minimally invasive and open EG surgery has been safely re-introduced in centres serving populations significantly affected by COVID-19. Differing approaches to mitigations against COVID-19 resulted in no infections amongst patients. Only two healthcare workers tested positive for (self-limiting) COVID-19 during the study period. Further study is urgently needed to understand the minimum precautionary measures required to ensure patients and staff remain safe.

## Electronic supplementary material

Below is the link to the electronic supplementary material.

Supplementary material 1 (DOCX 21 kb)
