# Supplementary material for: Is Re-introducing Major Open and Minimally Invasive Surgery during COVID-19 Safe for Patients and Healthcare Workers? An International, Multi-centre Cohort Study in the Field of Oesophago-gastric Surgery
Source: Ann Surg Oncol. 2021 Apr 17;28(9):4816–26. doi: 10.1245/s10434-021-09885-0 (PMC8053024; doi:10.1245/s10434-021-09885-0)
Supplement: Supplementary file 1 — Supplementary material 1 (DOCX 21 kb) [file 10434_2021_9885_MOESM1_ESM.docx]

**Table 1.** Precautions taken to minimise the risk of COVID-19 infections amongst patients and medical staff

| **Precaution** |  | **Amsterdam** | **Bilbao** | **Brescia** | **Leuven** | **London** | **Madrid** | **Manchester** | **Milan** | **Verona** |
| --- | --- | --- | --- | --- | --- | --- | --- | --- | --- | --- |
| **Hospital** | COVID-free hospital | **⚫** | **⚫** | **⚫** | **⚫** | **⚫** | **⚫** | **⚫** | **⚫** | **⚫** |
|  | COVID-free area within hospital managing COVID patients | **⚫** | **⚫** | **⚫** | **⚫** | **⚫** | **⚫** | **⚫** |  | **⚫** |
|  | Admission to COVID-free ITU/HDU | **⚫** | **⚫** | **⚫** | **⚫** | **⚫** | **⚫** | **⚫** | **⚫** | **⚫** |
|  | Admission to COVID-free area within ITU/HDU managing COVID patients | **⚫** |  | **⚫** |  | **⚫** | **⚫** | **⚫** |  | **⚫** |
|  | Discharge from ITU to dedicated COVID-free ward | **⚫** | **⚫** | **⚫** | **⚫** | **⚫** | **⚫** | **⚫** |  | **⚫** |
| **Patient Screening** | Pre-op isolation | **⚫** | **⚫** | **⚫** | **⚫** | **⚫** | **⚫** | **⚫** | **⚫** | **⚫** |
|  | Pre-op PCR antigen testing | **⚫** | **⚫** | **⚫** | **⚫** | **⚫** | **⚫** | **⚫** | **⚫** | **⚫** |
|  | Pre-op CT chest to rule out occult COVID infection | **⚫** | **⚫** | **⚫** | **⚫** | **⚫** | **⚫** | **⚫** | **⚫** | **⚫** |
|  | Post-op isolation | **⚫** | **⚫** | **⚫** | **⚫** | **⚫** | **⚫** | **⚫** | **⚫** | **⚫** |
| **Staff screening** | Staff testing | **⚫** | **⚫** | **⚫** | **⚫** | **⚫** | **⚫** | **⚫** | **⚫** | **⚫** |
|  | Staff screening | **⚫** | **⚫** | **⚫** | **⚫** | **⚫** | **⚫** | **⚫** | **⚫** | **⚫** |
| **PPE in the Operating Room** | *Double glove* | **⚫** | **⚫** | **⚫** | **⚫** | **⚫** | **⚫** | **⚫** | **⚫** | **⚫** |
|  | *Masks* | Surgical mask | FFP3  FFP2  Surgical mask | FFP3 | FFP3 | FFP3 | FFP2  Double surgical mask | FFP3 | FFP3  FFP2 | FFP3  Surgical mask |
|  | *Closed safety glasses* | **⚫** | **⚫** | **⚫** | **⚫** | **⚫** | **⚫** | **⚫** | **⚫** | **⚫** |
|  | *Visor* | **⚫** | **⚫** | **⚫** | **⚫** | **⚫** | **⚫** | **⚫** | **⚫** | **⚫** |
|  | *Respirator Hoods* | **⚫** | **⚫** | **⚫** | **⚫** | **⚫** | **⚫** | **⚫** | **⚫** | **⚫** |
|  | Balloon ports | **⚫** | **⚫** | **⚫** | **⚫** | **⚫** | **⚫** | **⚫** | **⚫** | **⚫** |
|  | Dedicated CO2 management system. | **⚫** | **⚫** | **⚫** | **⚫** | **⚫** | **⚫** | **⚫** | **⚫** | **⚫** |

⚫ Used routinely during the study ⚫ Not used during the study ⚫ Used selectively during the study. PPE (personal protective equipment)
